# Retraction Note: Structural simulation of adenosine phosphate via plumbagin and zoledronic acid competitively targets JNK/Erk to synergistically attenuate osteoclastogenesis in a breast cancer model

**DOI:** 10.1038/s41419-019-1606-1

**Published:** 2019-05-08

**Authors:** H. Qiao, T. Y. Wang, Z. F. Yu, X. G. Han, X. Q. Liu, Y. G. Wang, Q. M. Fan, A. Qin, T. T. Tang

**Affiliations:** 1 Shanghai Key Laboratory of Orthopaedic Implants, Department of Orthopaedic Surgery, Shanghai Ninth People’s Hospital, Shanghai Jiao Tong University School of Medicine, Shanghai, China; 2 Shanghai Key Laboratory of Orthopaedic Implants, Department of Pharmacy, Shanghai Ninth People’s Hospital, Shanghai Jiao Tong University School of Medicine, Shanghai, China

**Retraction Note to:**

**Cell Death and Disease** (2016)7:e2094

10.1038/cddis.2016.11 published online 11 February 2016

This article has been retracted at the request of the authors. After publication, the authors found that in Fig. 2B-a the first two images in the third row partly overlapped and that there is also overlap between the fourth and fifth images in the second row. The two images were taken from two adjacent wells, treated by ZA 0.3 μM-CM or ZA 0.75 μM-CM, with or without PL 1.25 μM. This overlap may have been caused by mishandling in the imaging process when the authors made microscope observations and so the findings are no longer reliable. All authors agree to this retraction.

